# Antibodies Targeting Two Epitopes in SARS-CoV-2 Neutralize Pseudoviruses with the Spike Proteins from Different Variants

**DOI:** 10.3390/pathogens10070869

**Published:** 2021-07-09

**Authors:** Chee-Hing Yang, Hui-Chun Li, Wen-Han Lee, Shih-Yen Lo

**Affiliations:** 1 Department of Laboratory Medicine and Biotechnology, Tzu Chi University, Hualien 97004, Taiwan; cheehing2@gms.tcu.edu.tw (C.-H.Y.); 107312152@gms.tcu.edu.tw (W.-H.L.); 2 Department of Biochemistry, Tzu Chi University, Hualien 97004, Taiwan; huichun@gms.tcu.edu.tw; 3 Department of Laboratory Medicine, Buddhist Tzu Chi General Hospital, Hualien 97004, Taiwan

**Keywords:** COVID-19, SARS-CoV-2, spike protein, epitope, D614G, Alpha variant, Beta variant, neutralization, pseudovirus

## Abstract

The COVID-19 pandemic was caused by SARS-CoV-2 infection. To prevent the spread of SARS-CoV-2, an effective vaccine is required. Two linear peptides from potential B-cell epitopes in the spike protein of SARS-CoV-2 (a.a. 440–460; a.a. 494–506) were synthesized and used to immunize rabbits. High-titer antibodies of IgG were produced, purified, and verified by Western blot analysis. Antibodies against these two epitopes could effectively neutralize SARS-CoV-2 pseudoviral particles with the spike proteins from not only the original strain (basal; wild-type), but also a strain with a single point mutation (D614G), and two other emerging variants (the Alpha and Beta variants) prevalent around the world, but not from SARS-CoV. In conclusion, antibodies against these two epitopes are protective. This information is important for the development of vaccines against SARS-CoV-2.

## 1. Introduction

Severe acute respiratory syndrome coronavirus 2 (SARS-CoV-2)—a novel human pathogen—is responsible for the ongoing global coronavirus disease 2019 (COVID-19) pandemic [1,2]. COVID-19 originated in the city of Wuhan, China [3]. Patients of COVID-19 experience symptoms ranging from fever, cough, and myalgia to severe respiratory failure [4]. SARS-CoV-2 is believed to have been acquired from a zoonotic source, and spreads through direct and/or contact transmission [2]. The world health organization (WHO) reported that more than 139 million confirmed cases of COVID-19, including 2,992,193 deaths, have occurred as of 17 April 2021 (https://covid19.who.int/ accessed on 28 June 2021).

SARS-CoV-2, an enveloped virus with a positive-sense single-stranded 29.9 kb RNA genome, is a member of the Coronaviridae family, which also includes the SARS-CoV and Middle East respiratory syndrome (MERS) viruses [1]. The SARS-CoV-2 genome comprises 15 open reading frames (ORFs) encoding at least 29 viral proteins [1]. Soon after the outbreak of SARS-CoV-2, six major clades were identified [5]. Among these clades, the D614G clade has become a dominant strain type since December 2019 [5]. Later, two different variants (B.1.1.7 and B.1.351) became prevalent around the world [6,7,8,9,10]. In collaboration with national authorities, institutions, and researchers, the WHO routinely assesses whether SARS-CoV-2 variants alter transmission or disease characteristics, or impact vaccines, therapeutics, diagnostics, or public health and social measures to control disease spread. To date, four variants have been identified: the Alpha variant (B.1.1.7 or GR/501Y.V1) [11], Beta variant (B.1.351 or GH/501Y.V2) [10,12], Gamma variant (P.1 or GR/501Y.V3), and Delta variant (B.1.617.2 or G/478K.V1) (https://www.who.int/publications/m/item/weekly-epidemiological-update-on-covid-19---22-june-2021/Accessed on 28 June 2021). The Beta variant—associated with a faster spread in countries like the United Kingdom and Australia [6]—escaped neutralization by most neutralizing monoclonal antibodies tested in a previous study [13]. Two major concerns related to another variant (Alpha) are the unprecedented number of mutations and the speed of spread [6].

To curb the spread of SARS-CoV-2 infections, rapid and accurate diagnostic tests are required for efficient screening and treatment of COVID-19 patients [14]. The gold standard diagnostic test for SARS-CoV-2 detection is reverse transcription quantitative polymerase chain reaction (RT-qPCR) using samples from nasopharyngeal swabs, throat swabs, or saliva [15]. At present, management of COVID-19 patients is mainly by supportive therapy, since no specific antiviral agent is available [1]. Thus, effective vaccines against SARS-CoV-2 to reduce the number of infections are critical [16,17]. SARS-CoV-2 can enter host cells by binding to the angiotensin-converting enzyme 2 (ACE2) through the viral spike protein [18]. All of the vaccines currently used to prevent SARS-CoV-2 infection contain or encode the spike protein [19,20]. To prevent SARS-CoV-2 infection, antibodies raised against the spike protein of the vaccine strain should be able to neutralize infection by any of the different SARS-CoV-2 variants. The emerging SARS-CoV-2 variants have in common a higher transmissibility—probably as a result of higher affinity of the spike protein for the cellular ACE2 receptor [21]. These SARS-CoV-2 variants may escape from the neutralizing antibodies of the vaccine strain [13], possibly through the mutations located in the B-cell epitopes of the spike protein. To address this issue, antibodies against two B-cell linear epitopes in the spike protein of the original clade (i.e., basal; wild-type) were raised. Neutralization tests of pseudoviruses with the spike proteins from different variants were conducted using these antibodies.

## 2. Results

### 2.1. Sequence Comparison of Two Potential B-Cell Epitopes Among Different SARS-CoV-2 Variants

**Figure 1:**
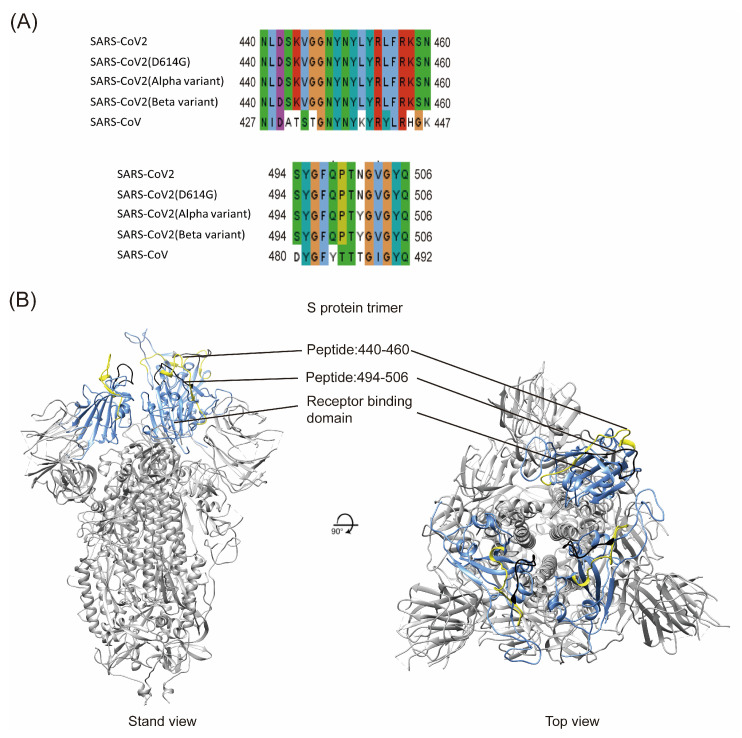
(**A**) Amino acid comparison of two linear peptides in the spike protein between different SARS-CoV-2 variants and SARS-CoV. (Upper) Amino acid comparison of a.a. 440–460 in the spike protein of different SARS-CoV-2 variants and a.a. 427–447 in the SARS-CoV spike protein; (Bottom) Amino acid comparison of a.a. 494–506 in the spike protein of different SARS-CoV-2 variants and a.a. 480–492 in the SARS-CoV spike protein. (**B**) Predicted localization of these two peptides in the RBD of the spike protein. RBD (blue); a.a. 440–460 (yellow); and a.a. 494–506 (black).

Potential T-cell and B-cell epitopes of SARS-CoV-2 have been identified in several previous studies using computational tools, machine learning, and immunological studies [22,23,24,25,26,27]. Among these epitopes, two potential neutralizing B-cell epitopes (a.a. 440–460; a.a. 494–506) were identified near the receptor-binding domain (RBD) of the spike proteins from both SARS-CoV-2 and SARS-CoV (https://doi.org/10.1101/2020.02.19.955484, accessed on 28 June 2021). Sequences of these two linear epitopes from the original strain (SARS-CoV-2 Wuhan-hu-1; basal; wild-type), three other different variants (D614G; Alpha variant; Beta variant) prevalent around the world (https://www.cdc.gov/coronavirus/2019-ncov/cases-updates/variant-surveillance/variant-info.html#Concern/ accessed on 28 June 2021), and SARS-CoV were compared (Figure 1). Four SARS-CoV-2 variants have identical sequences of a.a. 440–460, with 11 amino acid (a.a.) differences from SARS-CoV (Figure 1A, upper panel). For peptide 494–506, the Alpha and Beta variants have only one a.a. residue (a.a. 501) different from the basal and D614G variants, and five a.a. differences from SARS-CoV (Figure 1A, bottom panel). Thus, these two potential B-cell linear epitopes are rather conserved among different SARS-CoV-2 variants. The localization of these two peptides in the receptor-binding domain (RBD) of the spike protein was also predicted (Figure 1B).

### 2.2. Verification of Antibody Production by Western Blot Analysis

**Figure 2:**
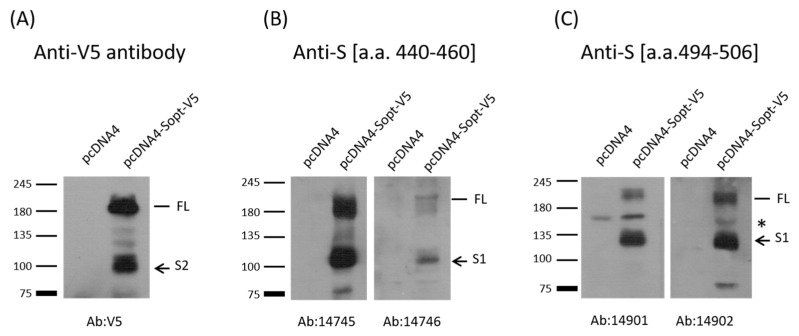
Western blot analysis of SARS-CoV-2 spike protein with V5 tag at its C-terminus using samples from Vero cells transiently transfected with either vector or with the plasmid-expressing full-length spike protein, which will be cleaved into S1 and S2 in the cells [28], as indicated. (**A**) Antibody against V5 tag could detect full-length (FL) and cleaved S2 of the spike protein with the V5 tag at its C-terminus [18]. (**B**) Anti-S (a.a. 440–460) antibodies (14745 and 14746) could react with full-length (FL) and cleaved S1 of the spike protein. (**C**) Anti-S (a.a. 494–506) (14901 and 14902) could react with full-length (FL) and cleaved S1 of the spike protein. A nonspecific band between FL and S1, marked as *, could react with Anti-S (a.a. 494–506).

To ascertain whether these two peptides (a.a. 440–460; a.a. 494–506) are B-cell epitopes or not, the two peptides from the SARS-CoV-2 basal strain were synthesized and used to immunize rabbits (two rabbits per peptide). Antibody generation was verified using Western blot analysis (Figure 2). For comparison, mock-vaccinated/purified rabbit IgG (1000-fold dilution of 5 mg/mL) and pre-immunized sera (1000-fold dilution of 4–10 mg/mL) were used as the controls. None of these sera reacted with the SARS-CoV-2 spike protein (data not shown). As expected, rabbit sera against either peptide could recognize full-length and one cleaved fragment (S1) of SARS-CoV-2 spike protein (Figure 2B and C). In contrast, anti-V5 tag reacted with full-length and one cleaved fragment (S2) of SARS-CoV-2 spike protein because V5 tag was fused to the C-terminus of the spike protein (Figure 2A). Thus, these two peptides are indeed B-cell epitopes. However, it is not known whether these two B-cell epitopes are exposed on the virus independently of other mutations in the spike proteins of different variants.

### 2.3. Neutralization Test of Pseudoviruses with the Spike Proteins from Different Variants

**Figure 3:**
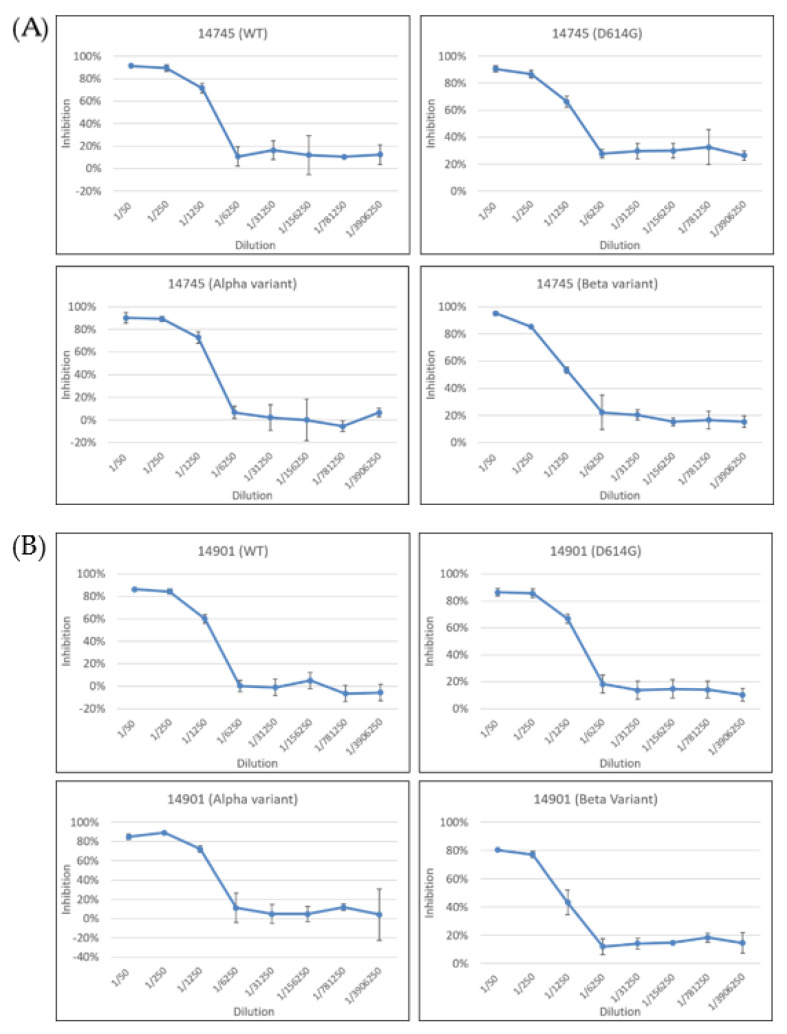
Neutralization test of pseudoviruses with spike proteins from different SARS-CoV-2 variants. WT (wild-type; basal). (**A**) Antibody 14745 against a.a. 440–460 could prevent infection by four different pseudoviruses when the dilution was less than 250-fold. (**B**) Antibody 14901 against a.a. 494–506 could prevent infection by four different pseudoviruses when the dilution was less than 250-fold.

To verify that the antibodies against these two peptides are protective, a neutralization test was conducted. One rabbit serum (14745) against a.a. 440–460, and another (14902) against a.a. 494–506, were chosen to perform the test, because they demonstrated better immune responses via Western blot (Figure 2). Pseudoviruses with the spike proteins from different SARS-CoV-2 variants—including the basal strain, single point mutation D614G strain, and two other different variants prevalent around the world (the Alpha and Beta variants)—were used as the challenging viruses. For comparison, mock-vaccinated/purified rabbit IgG (1000-fold dilution of 5 mg/mL) and pre-immunized sera (1000-fold dilution of 4–10 mg/mL) were used as the controls. None of these sera could neutralize the infection of the basal strain (data not shown). In contrast, antibody 14745 could neutralize the infection of these four different pseudoviruses when the dilution was less than 250-fold (i.e., when the antibody concentration was higher than 9.8 ug/mL) (Figure 3A). The half-maximal inhibition concentration (IC_50_) of Ab 14745 for the wild-type, D614G, Alpha, and Beta variants was 1.7, 1.8, 1.1, and 2.3 ug/mL, respectively. This is not surprising, since the sequence of a.a. 440–460 in these four variants is identical (Figure 1A). Antibody 14902 could also neutralize the infection of these four different pseudoviruses when the dilution was less than 250-fold (i.e., when the antibody concentration was higher than 9.76 ug/mL) (Figure 3B). The IC_50_ of Ab 14902 for the wild-type, D614G, Alpha, and Beta variants was 1.5, 1.3, 1.1, and 2.1 ug/mL, respectively. It was noted that the Alpha and Beta variants, unlike the D614G strain, have one a.a. residue (a.a. 501) different from that of the basal variant (Figure 1A). Thus, these two B-cell epitopes are still exposed on the virus independently of other mutations in the spike protein of the Alpha and Beta variants.

**Figure 4:**
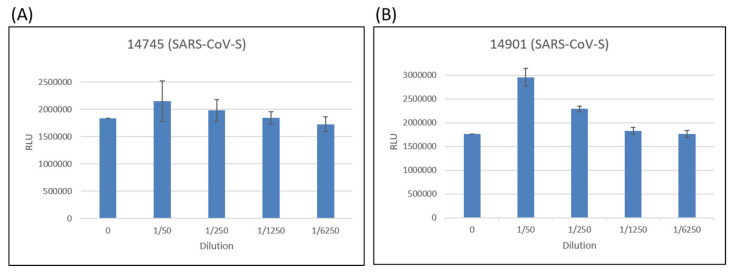
Neutralization test of pseudoviruses with SARS-CoV spike proteins. (**A**) Antibody 14745 could not prevent infection by pseudoviruses with SARS-CoV spike proteins in different antibody concentrations. (**B**) Antibody 14901 could not prevent infection by pseudoviruses with SARS-CoV spike proteins in different antibody concentrations.

For comparison, a neutralization test using pseudoviruses with SARS-CoV spike proteins was conducted. Neither of these two purified polyclonal antibodies could prevent infection (Figure 4). Thus, the 11 amino acid (a.a.) differences within the epitope a.a. 440–460, and the 5 a.a. differences within the epitope a.a. 494–506, did affect the immune responses.

## 3. Discussion

Two potential B-cell linear epitopes (a.a. 440–460 and a.a. 494–506) were found to be conserved among different SARS-CoV-2 variants (Figure 1A). These two peptides could generate an immune response successfully (Figure 2), indicating that they are B-cell epitopes. Antibodies against these two peptides could neutralize the pseudoviruses with spike proteins from different SARS-CoV-2 variants (Figure 3) (more than 90% in 14745 and more than 80% in 14901), indicating that they are protective. This information should be important, since the Beta variant has been reported to escape neutralization by most neutralizing monoclonal antibodies in a previous study [13].

An effective vaccine is needed to bring an end to the SARS-CoV-2 pandemic [19,29]. As expected, different approaches to developing vaccines against SARS-CoV-2 have been tested at unprecedented speed [30,31,32,33]. Among them, full-length or RBDs of the spike protein are promising vaccine candidates to induce neutralizing Abs [17]. However, autoantibodies have been found in critically ill COVID-19 patients [34]. Molecular mimicry has been proposed to explain this autoimmune response [35,36,37,38,39]; thus, a vaccine may generate an autoimmune response if it contains amino acid sequences similar to those of cellular proteins [40]. To avoid this problem, vaccines containing virus- but not cellular-specific epitopes are needed [26,27]. Moreover, SARS-CoV-2—a positive-stranded virus—could generate various mutants [41]. Identification of the protective B-cell epitopes within the spike protein could help to design vaccines; thus, the results in this study should be informative.

Antibodies against the two epitopes (a.a. 440–460 and a.a. 494–506) could neutralize SARS-CoV-2 pseudoviral particles with spike proteins from four different SARS-CoV-2 variants (basal; D614G; Alpha variant; Beta variant) (Figure 3), but not from SARS-CoV (Figure 4). These results suggest that vaccines against the basal strain may not be effective if further amino acid mutations are generated within these two linear epitopes. More coordinated regional or global efforts to monitor the emerging SARS-CoV-2 variants are needed.

## 4. Materials and Methods

### 4.1. Plasmid Construction

The expression plasmids used in this study were constructed using standard protocols, as described in our previous studies [42]. The PCR primer sequences used for cloning are available upon request. Renilla luciferase expression plasmid pLAS3w-Rluc was cloned by using PCR to amplify the RLuc sequence from pRL-TK (Promega, Madison, WI, USA) into pLAS3w-pPuro vector (RNAi core, Taipei City, Taiwan). SARS-CoV-2 spike-protein-expressing plasmid was constructed using the chemically synthesized codon-optimized sequence for expression in human cells [43], while the SARS-CoV S gene was derived from pcDNA3.1-S [42]. Human ACE2-expressing plasmid was constructed by cloning the ACE2 sequence from pcDNA3.1+/C-(K)DYK-ACE2 (GenScript) into pcDNA3.1-V5-HisA.

### 4.2. Cell Culture

Human embryonic kidney (HEK-293T; ATCC^®^ CRL-3216TM) cells were obtained from the American Type Culture Collection (ATCC). HEK293T cells were cultured in Dulbecco’s modified Eagle’s medium (DMEM; Gibco, Carlsbad, CA, USA) containing 10% fetal bovine serum (FBS), 100 U/mL penicillin, and 100 µg/mL streptomycin (Gibco). HEK-293T-ACE2 cells were generated via transduction of VSV-G pseudotyped lentivirus carrying the human ACE2 gene. Vero E6 cells were cultured in RPMI 1640 (Gibco) containing 10% FBS, 100 U/mL penicillin, and 100 µg/ mL streptomycin. All cultured cells were maintained at 37 °C with 5% CO_2_.

### 4.3. Antibody Production

Custom-designed rabbit polyclonal antibodies were obtained from ABclonal Technology (Wuhan, China). Two linear peptides (440′-NLDSKVGGNYNYLYRLFRKSN-460′, 494′-SYGFQPTNGVGYQ-506′) were synthesized, conjugated with KLH (keyhole limpet hemocyanin), and used to immunize New Zealand white rabbits (two rabbits per peptide). In brief, primary injection of KLH-conjugated peptides with CFA (complete Freund’s adjuvant), followed by two booster doses of KLH-conjugated peptides with IFA (incomplete Freund’s adjuvant), were conducted. Sera from the rabbits with three injections were collected and purified by affinity chromatography. The stock antibody concentrations used in this study were 2.45 mg/mL (14745) and 2.44 mg/mL (14901).

### 4.4. Protein Expression and Western Blot Analysis

Vero cells were transfected with empty vector or expressing plasmid for the full-length spike protein with V-5 tag at its C-terminus, as indicated. Forty-eight hours after transfection, protein lysates from these cells were collected and analyzed with SDS-PAGE, followed by Western blot analysis. Our previous procedures were followed for Western blot analysis [44]. The primary antibodies used for the analyses in this study were antibodies against V5 tag (Bio-Rad, Hercules, CA, USA) or peptides (a.a. 440–460; a.a. 494–506). Anti-rabbit IgG with HRP labeled was used as a secondary antibody.

### 4.5. Production and Purification of Pseudoviruses

The pseudotyped lentiviruses carrying spike proteins from four different SARS-CoV-2 variants were generated by transiently transfecting HEK-293T cells with pCMV-ΔR8.91-, pLAS2w.Fluc.Ppuro-, and pcDNA3.1-expressing plasmids with different S genes. The pLAS2w.Rluc.Ppuro plasmid was used to replace pLAS2w.Fluc.Ppuro for the generation of pseudoviruses with SARS-CoV spike proteins. HEK-293T cells were seeded one day before transfection, and the indicated plasmids were delivered into cells by using TransITR-LT1 transfection reagent (Mirus, Madison, WI, USA). The culture medium was refreshed at 16 h and harvested at 48 h and 72 h post-transfection. Cell debris was removed by centrifugation at 4000× *g* for 10 min, and the supernatant was passed through a 0.45-µm syringe filter (Pall Corporation, Port Washington, NY, USA). The pseudotyped lentiviruses were aliquoted and then stored at −80 °C [45].

### 4.6. Estimation of Lentiviral Titer by alamarBlue Assay 

The transduction unit (TU) of the SARS-CoV-2-pseudotyped lentivirus was estimated via cell viability assay in response to the limited dilution of the lentivirus. In brief, HEK-293T cells stably expressing the human ACE2 gene were plated on 96-well plates one day before lentivirus transduction. For the titering of pseudotyped lentivirus, different amounts of lentivirus were added into the culture medium containing polybrene (final concentration 8 µg/mL). Spin infection was carried out at 1100× *g* in 96-well plates for 30 min at 37 °C. After incubating cells at 37 °C for 16 h, the culture medium containing virus and polybrene were removed and replaced with complete DMEM containing 2.5 µg/mL puromycin. After treating puromycin for 48 h, the culture medium was removed and the cell viability was detected by using 10% alamarBlue reagents, according to the manufacturer’s instructions (Thermo Fisher Scientific, Waltham, MA, USA). The survival rate of uninfected cells (without puromycin treatment) was set as 100%. The virus titer (transduction units; TU) was determined by plotting the surviving cells versus the diluted viral dose [45].

### 4.7. Pseudotyped Lentivirus Neutralization Assay 

For the virus neutralization assay, heat-inactivated sera were serially diluted to the desired degree and incubated with 1000 TU of SARS-CoV-2-pseudotyped lentivirus for 1 h at 37 °C. The mixture was then inoculated with 10,000 HEK-293T cells stably expressing the human ACE2 gene in 96-well plates. The fresh medium was replaced at 16 h post-infection, and cells were continuously cultured for another 48 h. Then, the luciferase activity was determined using the Bright-Glo™ Luciferase Assay System (Promega). The relative light units (RLUs) were detected using Tecan i-control (Infinite 500). The percentage of inhibition was calculated as the ratio of RLU reduction in the presence of diluted serum to the RLU value of no serum control, and the equation used was as follows: (RLU _Control_ − RLU _Serum_)/RLU _Control_. The half-maximal inhibition concentration (IC_50_) was also calculated (https://www.aatbio.com/tools/ic50-calculator, accessed on 28 June 2021) [45].

### 4.8. Model Building and Peptide Mapping

The SARS-CoV-2 S PDBID:6VSB was used as the starting model. The localization of these two peptides (a.a. 440–460; a.a. 494–506) within the RBD was labeled using UCSF Chimera (V1.15) [46].

## 5. Conclusions

Two linear peptides (a.a. 440–460 and a.a. 494–506) in the spike protein of SARS-CoV-2 are neutralizing B-cell epitopes. Antibodies purified from the post-immune sera against these peptides from rabbits effectively neutralized the infection of SARS-CoV-2 pseudoviruses—even with one a.a. (N501Y) mutation—to more than 80%.

## Data Availability

No such data.
